# 1-(4-*tert*-Butyl­benz­yl)-3-(3,4,5-tri­methoxy­benz­yl)benzimidazolium bromide monohydrate

**DOI:** 10.1107/S1600536808043250

**Published:** 2008-12-24

**Authors:** Hakan Arslan, Don VanDerveer, Serpil Demir, İsmail Özdemir, Bekir Çetinkaya

**Affiliations:** a Department of Natural Sciences, Fayetteville State University, Fayetteville, NC 28301, USA; b Department of Chemistry, Faculty of Pharmacy, Mersin University, Mersin, TR 33169, Turkey; c Department of Chemistry, Clemson University, Clemson, SC 29634, USA; d Department of Chemistry, Faculty of Science and Arts, İnönü University, Malatya, TR 44280, Turkey; e Department of Chemistry, Faculty of Science, Ege University, Bornova-İzmir, TR 35100, Turkey

## Abstract

A novel *N*-heterocyclic carbene derivative, C_28_H_33_N_2_O_3_^+^·Br^−^·H_2_O, was synthesized and characterized by elemental analysis, ^1^H and ^13^C-NMR and IR spectroscopy and a single-crystal X-ray diffraction study. Ions of the title compound are linked by π⋯π stacking inter­actions (face–face separation 3.441 Å) and C—H⋯Br and O—H⋯Br inter­actions. Intra- and intermolecular C—H⋯O inter­actions are also present. The C—N bond lengths for the compound [1.329 (3), 1.325 (3), 1.389 (3) and 1.391 (3) Å] are all shorter than the average single C—N bond length of 1.48 Å, thus showing varying degrees of double-bond character.

## Related literature

For the synthesis, see: Yaşar *et al.* (2008 ▶). For general background, see: Herrmann (2002 ▶); Arduengo & Krafczyc (1998 ▶); Herrmann *et al.* (1995 ▶, 1998 ▶); Navarro *et al.* (2006 ▶). For related compounds, see: Yaşar *et al.* (2008 ▶); Arslan *et al.* (2009 ▶ and references therein). For bond-length data, see: Allen *et al.* (1987 ▶).
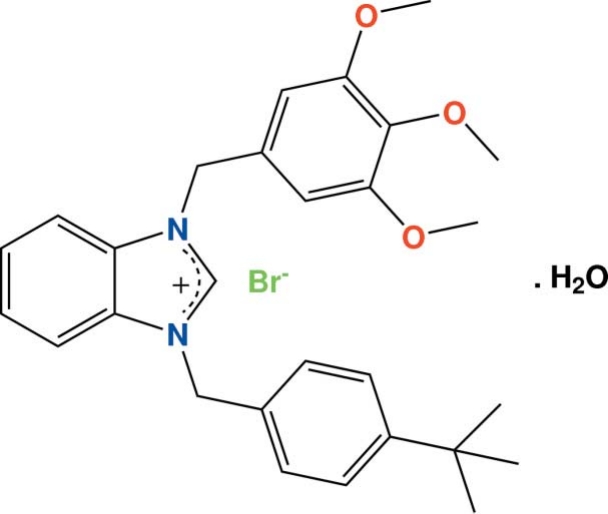


## Experimental

### 

#### Crystal data

C_28_H_33_N_2_O_3_^+^·Br^−^·H_2_O
*M* *_r_* = 543.49
Triclinic, 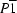

*a* = 10.389 (2) Å
*b* = 10.436 (2) Å
*c* = 14.038 (3) Å
α = 109.79 (3)°
β = 90.70 (3)°
γ = 103.57 (3)°
*V* = 1385.1 (6) Å^3^
*Z* = 2
Mo *K*α radiation
μ = 1.52 mm^−1^
*T* = 298 (2) K
0.48 × 0.29 × 0.26 mm

#### Data collection

Mercury CCD diffractometer
Absorption correction: multi-scan (*REQAB*; Jacobson, 1998 ▶) *T*_min_ = 0.514, *T*_max_ = 0.673
11938 measured reflections
4860 independent reflections
3921 reflections with *I* > 2σ(*I*)
*R*_int_ = 0.022

#### Refinement

*R*[*F* ^2^ > 2σ(*F* ^2^)] = 0.042
*wR*(*F* ^2^) = 0.112
*S* = 1.08
4860 reflections
328 parameters
2 restraints
H atoms treated by a mixture of independent and constrained refinement
Δρ_max_ = 0.31 e Å^−3^
Δρ_min_ = −0.55 e Å^−3^

### 

Data collection: *CrystalClear* (Rigaku/MSC, 2001 ▶); cell refinement: *CrystalClear*; data reduction: *CrystalClear*; program(s) used to solve structure: *SHELXTL* (Sheldrick, 2008 ▶); program(s) used to refine structure: *SHELXTL*; molecular graphics: *SHELXTL* and *Mercury* (Macrae *et al.*, 2006 ▶); software used to prepare material for publication: *SHELXTL*.

## Supplementary Material

Crystal structure: contains datablocks I. DOI: 10.1107/S1600536808043250/hg2459sup1.cif

Structure factors: contains datablocks I. DOI: 10.1107/S1600536808043250/hg2459Isup2.hkl

Additional supplementary materials:  crystallographic information; 3D view; checkCIF report

## Figures and Tables

**Table 1:** Hydrogen-bond geometry (Å, °)

*D*—H⋯*A*	*D*—H	H⋯*A*	*D*⋯*A*	*D*—H⋯*A*
O4—H4*A*⋯Br1^i^	0.87 (3)	2.54 (3)	3.393 (3)	169 (5)
O4—H4*B*⋯Br1^ii^	0.88 (5)	2.52 (5)	3.399 (3)	176 (5)
C1—H1⋯Br1	0.96	2.65	3.587 (3)	165
C3—H3⋯O2^iii^	0.96	2.57	3.294 (4)	132
C6—H6⋯O4	0.96	2.38	3.305 (5)	161
C10—H10⋯O4	0.96	2.59	3.463 (5)	152
C14—H14⋯Br1	0.96	2.88	3.823 (3)	167
C18—H18*A*⋯Br1^iv^	0.96	2.82	3.718 (3)	155

Symmetry codes: (i) 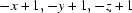; (ii) 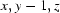; (iii) 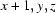; (iv) 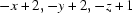.

**Fig. 1.**
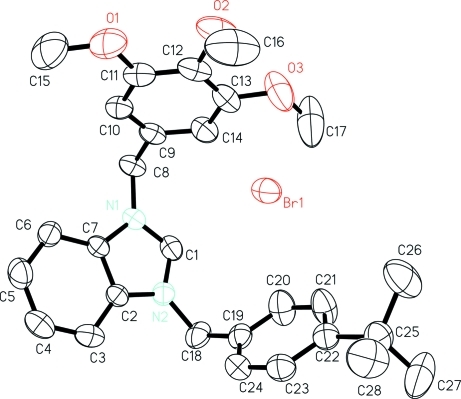
The molecular structure of the title compound, showing the atom-numbering scheme and displacement ellipsoids drawn at the 50% probability level.

**Fig. 2.**
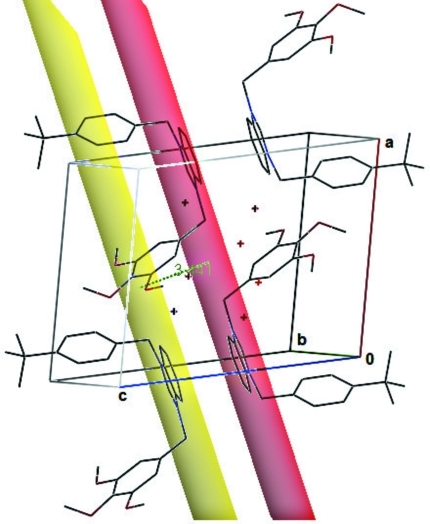
A packing diagram for (I).

**Fig. 3.**
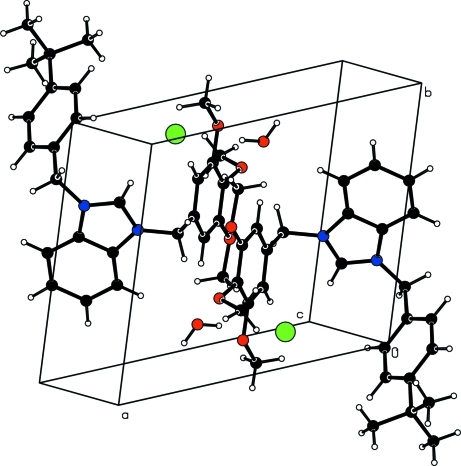
A packing diagram for (I).

**Fig. 4.**
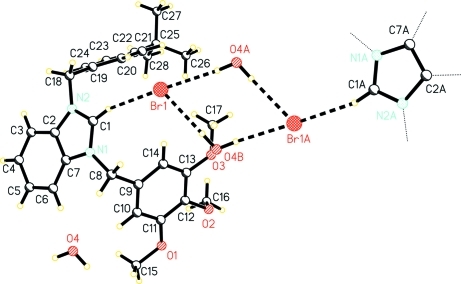
Hydrogen bonding for (I). Symmetry: O4A = x, 1 + y, z; O4B =1 - x,1 - y,1 - z; Br1A, etc. = 1 - x, 2 - y, 1 - z.

**Fig. 5.**
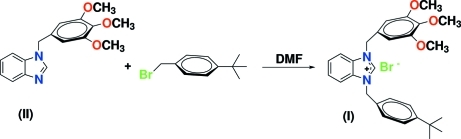
The preparation of the title compound.

## supplementary crystallographic information

### Comment

*N*-heterocyclic carbenes have attracted much interest as a new class of compound in organometallic chemistry. The applications of *N*-heterocyclic carbenes were first reported by Herrmann in 1998 (Herrmann *et al.*, 1998). Recently, Herrmann *et al.* and other researchers have designed new *N*-heterocyclic carbene compounds and have used them to prepare new catalysts for Suzuki-Miyura, Sonogashira, Stille and Heck reactions (Herrmann, 2002; Herrmann *et al.*, 1995; Navarro *et al*., 2006; Arduengo & Krafczyc, 1998).

Recently, we have focused on the synthesis, characterization and use of palladium, platinum and ruthenium *N*-heterocyclic carbene complexes as catalysts for Suzuki-Miyura and Heck reactions (Yaşar *et al.*, 2008; Arslan *et al.*, 2009, and references therein).

In the present work, we report the preparation and characterization of a novel *N*-heterocyclic carbene derivative, 1-(4-*tert*-butylbenzyl)-3-(3,4,5-trimethoxybenzyl)benzimidazolium bromide monohydrate, (I). The compound was purified by re-crystallization from ethanol:diethylether mixture (1:1) and characterized by elemental analysis, ^1^H and ^13^C-NMR and IR spectroscopy. The analytical and spectroscopic data are consistent with the proposed structure given in Scheme 1.

The molecular structure of the title compound is depicted in Figure 1. The crystal structure is composed of a 1-(4-*tert*-butylbenzyl)-3-(3,4,5-trimethoxybenzyl)benzimidazolium cation, a Br anion and solvent water molecules. All bond lengths in (I) are in normal ranges (Allen *et al.*,1987). The benzimidazole ring is almost coplanar with a maximum and a minimum deviation of 0.016 (2) Å for atom C2 and, 0.002 (2) Å for atom C6, respectively. In the crystal structure, π···π stacking interactions occurs between parallel benzimidazole rings, with a face-face separation of 3.441 Å (Figure 2) (Macrae *et al.*, 2006). The dihedral angle between the benzimidazole ring and 4-*tert*-butylbenzyl and 3,4,5-trimethoxybenzyl groups are 70.23 (3)° and 73.48 (3) *^o^*, respectively.

The C—N bond lengths for the investigated compound are all shorter than the average single C—N bond length of 1.48 Å, being N1—C1 = 1.329 (3) Å, N2—C1 =1.325 (3) Å, N1—C7 = 1.389 (3) Å, and N2—C2 = 1.391 (3) Å thus showing varying degrees of double bond character in these C—N bonds. The other CN bond lengths are in agreement with the expected 1.48 Å C—N single bond lengths. This information indicates a partial electron delocalization within the C7—N1—C1—N2—C2 fragment. The N1—C1—N2 bond angle is also consistent with this hypothesis.

The crystal packing is shown in Figure 3. The intermolecular C—H···Br and O—H···Br hydrogen bonds (Figure 4, Table 1) and π···π stacking interactions link the molecules of the title compound.

### Experimental

4-tertbutylbenzyl bromide (2.27 g, 10.0 mmol) was slowly added to a solution of 1-(3,4,5-trimethoxylbenzyl) benzimidazole (II) (2.98 g, 10.0 mmol) in DMF (5 mL) and the resulting mixture was stirred at room temperature for 5 h (Scheme 2). Diethylether (10 ml) was added to obtain a white crystalline solid which was filtered off. The solid was washed with diethylether (3 *x* 10 ml) dried under vacuum and the crude product was re-crystallized from ethanole/diethylether. *M*.p. = 246–247°C; yield 4.47 g, 85%; ν_(CN)_ = 1594 cm^-1. 1^H NMR (δ, 200.13 MHz, CDCl_3_): 1.25 (s, 9H, CH_2_C_6_H_4_C(C*H*_3_)_3_-*p*); 3.86 and 3.79 (s, 9H, CH_2_C_6_H_2_(OC*H*_3_)_3_-3,4,5); 5.81 (s,4*H*, C*H*_2_C_6_H_2_(OCH_3_)_3_-3,4,5 and C*H*_2_C_6_H_4_C(CH_3_)_3_-*p*); 6.90 (s, 2H, CH_2_C_6_*H*_2_(OCH_3_)_3_-3,4,5); 7.33 and 7.48 (m, 8H, NC_6_*H*_4_N and CH_2_C_6_*H*_4_C(CH_3_)_3_-*p*); 11.64 (s, 1H, NC*H*N). ^13^C NMR (δ, 50 MHz, CDCl_3_): 31.6 (CH_2_C_6_H_4_C(*C*H_3_)_3_-*p*); 35.1 (CH_2_C_6_H_4_*C*(CH_3_)_3_-*p*); 51.6 (*C*H_2_C_6_H_4_C(CH_3_)_3_-*p*); 52.1 (*C*H_2_C_6_H_2_(OCH_3_)_3_-3,4,5); 57.2 and 61.2 (CH_2_C_6_H_2_(O*C*H_3_)_3_-3,4,5); 106.5, 131.7, 138.9 and 152.9 (CH_2_*C*_6_H_2_(OCH_3_)_3_-3,4,5); 114.2, 127.5, 128.7, 130.1, 131.8 and 143.2 (N*C*_6_H_4_N); 114.1, 126.7, 128.5 and 130.9 (CH_2_*C*_6_H_4_C(CH_3_)_3_-*p*); 154.2 (N*C*HN). Anal. Found: C, 63.96; H, 6.28; N: 5.35. Calc. for C_28_H_33_N_2_O_3_Br: C, 64.00; H, 6.33; N, 5.33.

### Refinement

All H atoms attached to carbons were geometrically fixed and allowed to ride on the corresponding non-H atom with C—H = 0.96 Å, and *U*_iso_(H) = 1.5*U*_eq_(C) of the attached C atom for methyl H atoms and 1.2*U*_eq_(C) for other H atoms. The water H atoms were located from a Fourier map and their distances were constrained to 0.86 Å and the *U*_iso_(H) = 1.5*U*_eq_(O).

### Crystal data

**Table:**

C_28_H_33_N_2_O_3_^+^·Br^−^·H_2_O	*Z* = 2
*M**_r_* = 543.49	*F*(000) = 568
Triclinic, *P*1	*D*_x_ = 1.303 Mg m^−^^3^
Hall symbol: -P 1	Mo *K*α radiation, λ = 0.71073 Å
*a* = 10.389 (2) Å	Cell parameters from 4659 reflections
*b* = 10.436 (2) Å	θ = 3.2–26.4°
*c* = 14.038 (3) Å	µ = 1.52 mm^−^^1^
α = 109.79 (3)°	*T* = 298 K
β = 90.70 (3)°	Rod, colorless
γ = 103.57 (3)°	0.48 × 0.29 × 0.26 mm
*V* = 1385.1 (6) Å^3^	

### Data collection

**Table:**

Mercury CCD diffractometer	4860 independent reflections
Radiation source: Sealed Tube	3921 reflections with *I* > 2σ(*I*)
Graphite Monochromator	*R*_int_ = 0.022
Detector resolution: 14.6306 pixels mm^-1^	θ_max_ = 25.0°, θ_min_ = 3.2°
ω scans	*h* = −12→12
Absorption correction: multi-scan (REQAB; Jacobson, 1998)	*k* = −12→12
*T*_min_ = 0.514, *T*_max_ = 0.673	*l* = −16→16
11938 measured reflections	

### Refinement

**Table:**

Refinement on *F*^2^	Primary atom site location: structure-invariant direct methods
Least-squares matrix: full	Secondary atom site location: difference Fourier map
*R*[*F*^2^ > 2σ(*F*^2^)] = 0.042	Hydrogen site location: inferred from neighbouring sites
*wR*(*F*^2^) = 0.112	H atoms treated by a mixture of independent and constrained refinement
*S* = 1.08	*w* = 1/[σ^2^(*F*_o_^2^) + (0.0505*P*)^2^ + 0.6557*P*] where *P* = (*F*_o_^2^ + 2*F*_c_^2^)/3
4860 reflections	(Δ/σ)_max_ = 0.001
328 parameters	Δρ_max_ = 0.31 e Å^−^^3^
2 restraints	Δρ_min_ = −0.55 e Å^−^^3^

### Special details

**Table:**

Geometry. All e.s.d.'s (except the e.s.d. in the dihedral angle between two l.s. planes) are estimated using the full covariance matrix. The cell e.s.d.'s are taken into account individually in the estimation of e.s.d.'s in distances, angles and torsion angles; correlations between e.s.d.'s in cell parameters are only used when they are defined by crystal symmetry. An approximate (isotropic) treatment of cell e.s.d.'s is used for estimating e.s.d.'s involving l.s. planes.
Refinement. Refinement of *F*^2^ against ALL reflections. The weighted *R*-factor *wR* and goodness of fit *S* are based on *F*^2^, conventional *R*-factors *R* are based on *F*, with *F* set to zero for negative *F*^2^. The threshold expression of *F*^2^ > σ(*F*^2^) is used only for calculating *R*-factors(gt) *etc*. and is not relevant to the choice of reflections for refinement. *R*-factors based on *F*^2^ are statistically about twice as large as those based on *F*, and *R*- factors based on ALL data will be even larger.

### Fractional atomic coordinates and isotropic or equivalent isotropic displacement parameters (Å^2^)

**Table:**

	*x*	*y*	*z*	*U*_iso_*/*U*_eq_	
Br1	0.75295 (3)	0.93707 (4)	0.51515 (3)	0.06917 (15)	
N1	0.8315 (2)	0.5830 (2)	0.57685 (15)	0.0384 (5)	
N2	1.0326 (2)	0.7189 (2)	0.62364 (16)	0.0407 (5)	
C1	0.9118 (3)	0.7064 (3)	0.5843 (2)	0.0439 (6)	
H1	0.8858	0.7766	0.5640	0.053*	
C2	1.0322 (2)	0.5974 (2)	0.64403 (18)	0.0363 (5)	
C3	1.1313 (3)	0.5558 (3)	0.6834 (2)	0.0497 (7)	
H3	1.2196	0.6159	0.7050	0.060*	
C4	1.0966 (3)	0.4233 (3)	0.6901 (2)	0.0609 (8)	
H4	1.1626	0.3902	0.7168	0.073*	
C5	0.9681 (3)	0.3364 (3)	0.6591 (2)	0.0573 (7)	
H5	0.9483	0.2449	0.6650	0.069*	
C6	0.8695 (3)	0.3772 (3)	0.6207 (2)	0.0449 (6)	
H6	0.7811	0.3170	0.5997	0.054*	
C7	0.9039 (2)	0.5104 (2)	0.61354 (18)	0.0351 (5)	
C8	0.6876 (2)	0.5379 (3)	0.5428 (2)	0.0454 (6)	
H8A	0.6617	0.4377	0.5084	0.054*	
H8B	0.6699	0.5814	0.4955	0.054*	
C9	0.6061 (2)	0.5776 (3)	0.6319 (2)	0.0438 (6)	
C10	0.5443 (3)	0.4785 (3)	0.6729 (2)	0.0456 (6)	
H10	0.5523	0.3835	0.6449	0.055*	
C11	0.4705 (3)	0.5178 (3)	0.7550 (2)	0.0521 (7)	
C12	0.4583 (3)	0.6547 (3)	0.7952 (2)	0.0537 (7)	
C13	0.5222 (3)	0.7534 (3)	0.7543 (2)	0.0553 (7)	
C14	0.5963 (3)	0.7155 (3)	0.6720 (2)	0.0508 (7)	
H14	0.6399	0.7839	0.6435	0.061*	
C15	0.4234 (4)	0.2906 (4)	0.7695 (3)	0.0873 (12)	
H15A	0.3873	0.2414	0.6998	0.131*	
H15B	0.3783	0.2416	0.8112	0.131*	
H15C	0.5166	0.2951	0.7756	0.131*	
C16	0.4304 (4)	0.7401 (6)	0.9699 (3)	0.1083 (17)	
H16A	0.4738	0.6735	0.9805	0.162*	
H16B	0.3620	0.7526	1.0150	0.162*	
H16C	0.4944	0.8286	0.9835	0.162*	
C17	0.5773 (7)	0.9936 (4)	0.7672 (4)	0.119 (2)	
H17A	0.6705	0.9984	0.7736	0.179*	
H17B	0.5622	1.0823	0.8082	0.179*	
H17C	0.5478	0.9731	0.6973	0.179*	
C18	1.1457 (3)	0.8450 (3)	0.6461 (2)	0.0536 (7)	
H18A	1.1451	0.8826	0.5925	0.064*	
H18B	1.2277	0.8182	0.6483	0.064*	
C19	1.1387 (3)	0.9570 (3)	0.7460 (2)	0.0450 (6)	
C20	1.0657 (4)	1.0516 (3)	0.7490 (2)	0.0671 (9)	
H20	1.0205	1.0466	0.6873	0.081*	
C21	1.0563 (4)	1.1546 (3)	0.8403 (2)	0.0677 (9)	
H21	1.0048	1.2198	0.8403	0.081*	
C22	1.1192 (3)	1.1659 (3)	0.9317 (2)	0.0452 (6)	
C23	1.1931 (3)	1.0704 (3)	0.9268 (2)	0.0532 (7)	
H23	1.2390	1.0752	0.9883	0.064*	
C24	1.2033 (3)	0.9667 (3)	0.8350 (2)	0.0533 (7)	
H24	1.2558	0.9019	0.8342	0.064*	
C25	1.1111 (3)	1.2836 (3)	1.0308 (2)	0.0591 (8)	
C26	0.9702 (4)	1.3046 (5)	1.0365 (3)	0.0903 (13)	
H26A	0.9665	1.3779	1.0993	0.135*	
H26B	0.9085	1.2186	1.0334	0.135*	
H26C	0.9472	1.3306	0.9804	0.135*	
C27	1.2089 (5)	1.4190 (4)	1.0328 (4)	0.1013 (15)	
H27A	1.2976	1.4066	1.0315	0.152*	
H27B	1.2037	1.4947	1.0937	0.152*	
H27C	1.1868	1.4408	0.9745	0.152*	
C28	1.1443 (4)	1.2485 (5)	1.1241 (3)	0.0840 (11)	
H28A	1.2353	1.2435	1.1266	0.126*	
H28B	1.0870	1.1594	1.1196	0.126*	
H28C	1.1315	1.3204	1.1846	0.126*	
O1	0.4055 (2)	0.4294 (3)	0.80195 (18)	0.0717 (6)	
O2	0.3740 (2)	0.6898 (3)	0.86948 (17)	0.0741 (7)	
O3	0.5058 (3)	0.8868 (3)	0.8002 (2)	0.0870 (8)	
O4	0.5832 (3)	0.1696 (3)	0.4946 (3)	0.0969 (10)	
H4A	0.499 (2)	0.144 (6)	0.501 (4)	0.145*	
H4B	0.623 (5)	0.107 (5)	0.501 (4)	0.145*	

### Atomic displacement parameters (Å^2^)

**Table:**

	*U*^11^	*U*^22^	*U*^33^	*U*^12^	*U*^13^	*U*^23^
Br1	0.0556 (2)	0.0784 (2)	0.0980 (3)	0.02072 (16)	0.01227 (17)	0.0592 (2)
N1	0.0361 (11)	0.0451 (11)	0.0394 (12)	0.0158 (9)	0.0067 (9)	0.0176 (9)
N2	0.0412 (12)	0.0386 (11)	0.0437 (12)	0.0106 (9)	0.0121 (9)	0.0156 (9)
C1	0.0482 (15)	0.0452 (14)	0.0470 (15)	0.0210 (12)	0.0139 (12)	0.0208 (12)
C2	0.0364 (12)	0.0393 (12)	0.0342 (13)	0.0132 (10)	0.0078 (10)	0.0117 (10)
C3	0.0378 (14)	0.0634 (17)	0.0469 (16)	0.0149 (13)	0.0004 (12)	0.0169 (13)
C4	0.0558 (18)	0.073 (2)	0.067 (2)	0.0308 (16)	−0.0007 (15)	0.0315 (16)
C5	0.067 (2)	0.0494 (16)	0.0648 (19)	0.0205 (15)	0.0037 (15)	0.0284 (14)
C6	0.0463 (15)	0.0391 (13)	0.0477 (15)	0.0082 (11)	0.0045 (12)	0.0148 (11)
C7	0.0348 (12)	0.0396 (12)	0.0330 (12)	0.0147 (10)	0.0060 (10)	0.0119 (10)
C8	0.0360 (13)	0.0599 (16)	0.0433 (15)	0.0190 (12)	0.0027 (11)	0.0173 (12)
C9	0.0329 (13)	0.0567 (15)	0.0432 (14)	0.0176 (12)	0.0004 (11)	0.0155 (12)
C10	0.0366 (13)	0.0535 (15)	0.0483 (16)	0.0120 (12)	0.0020 (11)	0.0195 (12)
C11	0.0363 (14)	0.0673 (18)	0.0546 (17)	0.0098 (13)	0.0039 (12)	0.0260 (14)
C12	0.0355 (14)	0.079 (2)	0.0471 (16)	0.0209 (14)	0.0077 (12)	0.0187 (14)
C13	0.0541 (17)	0.0633 (18)	0.0550 (17)	0.0314 (15)	0.0114 (14)	0.0175 (14)
C14	0.0493 (16)	0.0589 (17)	0.0537 (17)	0.0222 (13)	0.0107 (13)	0.0260 (13)
C15	0.091 (3)	0.081 (3)	0.099 (3)	0.006 (2)	0.019 (2)	0.054 (2)
C16	0.078 (3)	0.170 (5)	0.054 (2)	0.022 (3)	0.013 (2)	0.016 (3)
C17	0.195 (6)	0.063 (2)	0.112 (4)	0.055 (3)	0.059 (4)	0.030 (2)
C18	0.0514 (16)	0.0446 (15)	0.0592 (18)	0.0025 (12)	0.0187 (14)	0.0175 (13)
C19	0.0452 (14)	0.0382 (13)	0.0515 (16)	0.0060 (11)	0.0132 (12)	0.0184 (11)
C20	0.097 (3)	0.0610 (19)	0.0488 (18)	0.0340 (18)	−0.0027 (17)	0.0174 (14)
C21	0.097 (3)	0.0613 (19)	0.0550 (19)	0.0438 (19)	0.0010 (18)	0.0179 (15)
C22	0.0462 (15)	0.0448 (14)	0.0458 (15)	0.0110 (12)	0.0085 (12)	0.0176 (12)
C23	0.0498 (16)	0.0567 (17)	0.0518 (17)	0.0147 (13)	−0.0032 (13)	0.0170 (13)
C24	0.0456 (15)	0.0498 (15)	0.0653 (19)	0.0185 (13)	0.0051 (14)	0.0170 (14)
C25	0.0622 (19)	0.0573 (17)	0.0523 (18)	0.0180 (15)	0.0122 (15)	0.0103 (14)
C26	0.091 (3)	0.113 (3)	0.072 (3)	0.056 (3)	0.024 (2)	0.018 (2)
C27	0.127 (4)	0.053 (2)	0.093 (3)	0.002 (2)	0.024 (3)	0.000 (2)
C28	0.087 (3)	0.112 (3)	0.0454 (19)	0.032 (2)	0.0076 (18)	0.0146 (19)
O1	0.0604 (14)	0.0848 (16)	0.0758 (16)	0.0089 (12)	0.0203 (12)	0.0416 (13)
O2	0.0492 (12)	0.1158 (19)	0.0554 (14)	0.0316 (13)	0.0177 (10)	0.0202 (13)
O3	0.111 (2)	0.0785 (16)	0.0900 (19)	0.0592 (16)	0.0421 (16)	0.0286 (14)
O4	0.0533 (14)	0.0568 (14)	0.174 (3)	0.0082 (12)	−0.0014 (18)	0.0367 (17)

### Geometric parameters (Å, °)

**Table:**

N1—C1	1.329 (3)	C16—H16A	0.9599
N1—C7	1.389 (3)	C16—H16B	0.9599
N1—C8	1.478 (3)	C16—H16C	0.9599
N2—C1	1.325 (3)	C17—O3	1.407 (5)
N2—C2	1.391 (3)	C17—H17A	0.9599
N2—C18	1.481 (3)	C17—H17B	0.9599
C1—H1	0.9600	C17—H17C	0.9599
C2—C3	1.382 (4)	C18—C19	1.507 (4)
C2—C7	1.393 (3)	C18—H18A	0.9600
C3—C4	1.380 (4)	C18—H18B	0.9600
C3—H3	0.9600	C19—C20	1.370 (4)
C4—C5	1.394 (5)	C19—C24	1.372 (4)
C4—H4	0.9600	C20—C21	1.389 (4)
C5—C6	1.368 (4)	C20—H20	0.9600
C5—H5	0.9600	C21—C22	1.388 (4)
C6—C7	1.390 (3)	C21—H21	0.9600
C6—H6	0.9600	C22—C23	1.380 (4)
C8—C9	1.513 (4)	C22—C25	1.532 (4)
C8—H8A	0.9600	C23—C24	1.399 (4)
C8—H8B	0.9600	C23—H23	0.9600
C9—C10	1.383 (4)	C24—H24	0.9600
C9—C14	1.386 (4)	C25—C27	1.526 (5)
C10—C11	1.390 (4)	C25—C26	1.529 (5)
C10—H10	0.9600	C25—C28	1.530 (5)
C11—O1	1.369 (3)	C26—H26A	0.9599
C11—C12	1.385 (4)	C26—H26B	0.9599
C12—O2	1.380 (3)	C26—H26C	0.9599
C12—C13	1.385 (4)	C27—H27A	0.9599
C13—O3	1.374 (4)	C27—H27B	0.9599
C13—C14	1.392 (4)	C27—H27C	0.9599
C14—H14	0.9600	C28—H28A	0.9599
C15—O1	1.422 (5)	C28—H28B	0.9599
C15—H15A	0.9599	C28—H28C	0.9599
C15—H15B	0.9599	O4—H4A	0.87 (2)
C15—H15C	0.9599	O4—H4B	0.88 (5)
C16—O2	1.394 (5)		
			
C1—N1—C7	108.2 (2)	H16A—C16—H16C	109.5
C1—N1—C8	125.0 (2)	H16B—C16—H16C	109.5
C7—N1—C8	126.6 (2)	O3—C17—H17A	109.5
C1—N2—C2	108.4 (2)	O3—C17—H17B	109.5
C1—N2—C18	124.9 (2)	H17A—C17—H17B	109.5
C2—N2—C18	126.6 (2)	O3—C17—H17C	109.5
N2—C1—N1	110.5 (2)	H17A—C17—H17C	109.5
N2—C1—H1	124.8	H17B—C17—H17C	109.5
N1—C1—H1	124.8	N2—C18—C19	111.7 (2)
C3—C2—N2	132.0 (2)	N2—C18—H18A	109.3
C3—C2—C7	121.6 (2)	C19—C18—H18A	109.3
N2—C2—C7	106.3 (2)	N2—C18—H18B	109.3
C4—C3—C2	116.5 (3)	C19—C18—H18B	109.3
C4—C3—H3	121.7	H18A—C18—H18B	107.9
C2—C3—H3	121.7	C20—C19—C24	118.7 (3)
C3—C4—C5	121.6 (3)	C20—C19—C18	119.6 (3)
C3—C4—H4	119.2	C24—C19—C18	121.7 (3)
C5—C4—H4	119.2	C19—C20—C21	120.7 (3)
C6—C5—C4	122.1 (3)	C19—C20—H20	119.7
C6—C5—H5	118.9	C21—C20—H20	119.7
C4—C5—H5	118.9	C22—C21—C20	121.9 (3)
C5—C6—C7	116.5 (3)	C22—C21—H21	119.1
C5—C6—H6	121.8	C20—C21—H21	119.1
C7—C6—H6	121.8	C23—C22—C21	116.4 (3)
N1—C7—C6	131.8 (2)	C23—C22—C25	122.7 (3)
N1—C7—C2	106.6 (2)	C21—C22—C25	120.8 (3)
C6—C7—C2	121.6 (2)	C22—C23—C24	121.9 (3)
N1—C8—C9	111.2 (2)	C22—C23—H23	119.1
N1—C8—H8A	109.4	C24—C23—H23	119.1
C9—C8—H8A	109.4	C19—C24—C23	120.4 (3)
N1—C8—H8B	109.4	C19—C24—H24	119.8
C9—C8—H8B	109.4	C23—C24—H24	119.8
H8A—C8—H8B	108.0	C27—C25—C26	109.6 (3)
C10—C9—C14	120.8 (2)	C27—C25—C28	110.0 (3)
C10—C9—C8	120.6 (2)	C26—C25—C28	107.1 (3)
C14—C9—C8	118.6 (2)	C27—C25—C22	108.2 (3)
C9—C10—C11	119.5 (3)	C26—C25—C22	110.5 (3)
C9—C10—H10	120.3	C28—C25—C22	111.5 (3)
C11—C10—H10	120.3	C25—C26—H26A	109.5
O1—C11—C12	114.9 (3)	C25—C26—H26B	109.5
O1—C11—C10	124.7 (3)	H26A—C26—H26B	109.5
C12—C11—C10	120.4 (3)	C25—C26—H26C	109.5
O2—C12—C11	120.2 (3)	H26A—C26—H26C	109.5
O2—C12—C13	120.0 (3)	H26B—C26—H26C	109.5
C11—C12—C13	119.6 (3)	C25—C27—H27A	109.5
O3—C13—C12	115.3 (3)	C25—C27—H27B	109.5
O3—C13—C14	124.1 (3)	H27A—C27—H27B	109.5
C12—C13—C14	120.6 (3)	C25—C27—H27C	109.5
C9—C14—C13	119.1 (3)	H27A—C27—H27C	109.5
C9—C14—H14	120.5	H27B—C27—H27C	109.5
C13—C14—H14	120.5	C25—C28—H28A	109.5
O1—C15—H15A	109.5	C25—C28—H28B	109.5
O1—C15—H15B	109.5	H28A—C28—H28B	109.5
H15A—C15—H15B	109.5	C25—C28—H28C	109.5
O1—C15—H15C	109.5	H28A—C28—H28C	109.5
H15A—C15—H15C	109.5	H28B—C28—H28C	109.5
H15B—C15—H15C	109.5	C11—O1—C15	117.4 (3)
O2—C16—H16A	109.5	C12—O2—C16	116.4 (3)
O2—C16—H16B	109.5	C13—O3—C17	117.6 (3)
H16A—C16—H16B	109.5	H4A—O4—H4B	110 (5)
O2—C16—H16C	109.5		
			
C2—N2—C1—N1	−0.4 (3)	O2—C12—C13—O3	6.4 (4)
C18—N2—C1—N1	−177.7 (2)	C11—C12—C13—O3	−179.1 (3)
C7—N1—C1—N2	0.1 (3)	O2—C12—C13—C14	−173.2 (3)
C8—N1—C1—N2	175.6 (2)	C11—C12—C13—C14	1.4 (5)
C1—N2—C2—C3	179.0 (3)	C10—C9—C14—C13	−0.4 (4)
C18—N2—C2—C3	−3.8 (4)	C8—C9—C14—C13	−179.7 (3)
C1—N2—C2—C7	0.6 (3)	O3—C13—C14—C9	179.9 (3)
C18—N2—C2—C7	177.8 (2)	C12—C13—C14—C9	−0.5 (4)
N2—C2—C3—C4	−177.6 (3)	C1—N2—C18—C19	81.3 (3)
C7—C2—C3—C4	0.5 (4)	C2—N2—C18—C19	−95.5 (3)
C2—C3—C4—C5	−0.3 (4)	N2—C18—C19—C20	−86.2 (3)
C3—C4—C5—C6	−0.1 (5)	N2—C18—C19—C24	93.5 (3)
C4—C5—C6—C7	0.2 (4)	C24—C19—C20—C21	−0.5 (5)
C1—N1—C7—C6	−178.2 (3)	C18—C19—C20—C21	179.1 (3)
C8—N1—C7—C6	6.3 (4)	C19—C20—C21—C22	−0.3 (6)
C1—N1—C7—C2	0.3 (3)	C20—C21—C22—C23	0.8 (5)
C8—N1—C7—C2	−175.2 (2)	C20—C21—C22—C25	177.9 (3)
C5—C6—C7—N1	178.4 (3)	C21—C22—C23—C24	−0.6 (4)
C5—C6—C7—C2	0.0 (4)	C25—C22—C23—C24	−177.7 (3)
C3—C2—C7—N1	−179.1 (2)	C20—C19—C24—C23	0.7 (4)
N2—C2—C7—N1	−0.5 (2)	C18—C19—C24—C23	−178.9 (3)
C3—C2—C7—C6	−0.4 (4)	C22—C23—C24—C19	−0.1 (4)
N2—C2—C7—C6	178.2 (2)	C23—C22—C25—C27	98.5 (4)
C1—N1—C8—C9	−90.8 (3)	C21—C22—C25—C27	−78.4 (4)
C7—N1—C8—C9	84.0 (3)	C23—C22—C25—C26	−141.5 (3)
N1—C8—C9—C10	−100.3 (3)	C21—C22—C25—C26	41.6 (4)
N1—C8—C9—C14	78.9 (3)	C23—C22—C25—C28	−22.5 (4)
C14—C9—C10—C11	0.5 (4)	C21—C22—C25—C28	160.6 (3)
C8—C9—C10—C11	179.7 (2)	C12—C11—O1—C15	−175.0 (3)
C9—C10—C11—O1	−179.8 (3)	C10—C11—O1—C15	5.2 (5)
C9—C10—C11—C12	0.3 (4)	C11—C12—O2—C16	92.8 (4)
O1—C11—C12—O2	−6.6 (4)	C13—C12—O2—C16	−92.7 (4)
C10—C11—C12—O2	173.3 (3)	C12—C13—O3—C17	175.0 (4)
O1—C11—C12—C13	178.9 (3)	C14—C13—O3—C17	−5.5 (6)
C10—C11—C12—C13	−1.2 (4)		

### Hydrogen-bond geometry (Å, °)

**Table:**

*D*—H···*A*	*D*—H	H···*A*	*D*···*A*	*D*—H···*A*
O4—H4A···Br1^i^	0.87 (3)	2.54 (3)	3.393 (3)	169 (5)
O4—H4B···Br1^ii^	0.88 (5)	2.52 (5)	3.399 (3)	176 (5)
C1—H1···Br1	0.96	2.65	3.587 (3)	165
C3—H3···O2^iii^	0.96	2.57	3.294 (4)	132
C6—H6···O4	0.96	2.38	3.305 (5)	161
C10—H10···O4	0.96	2.59	3.463 (5)	152
C14—H14···Br1	0.96	2.88	3.823 (3)	167
C18—H18A···Br1^iv^	0.96	2.82	3.718 (3)	155

Symmetry codes: (i) −*x*+1, −*y*+1, −*z*+1; (ii) *x*, *y*−1, *z*; (iii) *x*+1, *y*, *z*; (iv) −*x*+2, −*y*+2, −*z*+1.
